# Selecting Reliable and Robust Freshwater Macroalgae for Biomass Applications

**DOI:** 10.1371/journal.pone.0064168

**Published:** 2013-05-22

**Authors:** Rebecca J. Lawton, Rocky de Nys, Nicholas A. Paul

**Affiliations:** School of Marine and Tropical Biology, James Cook University, Townsville, Queensland, Australia; Dowling College, United States of America

## Abstract

Intensive cultivation of freshwater macroalgae is likely to increase with the development of an algal biofuels industry and algal bioremediation. However, target freshwater macroalgae species suitable for large-scale intensive cultivation have not yet been identified. Therefore, as a first step to identifying target species, we compared the productivity, growth and biochemical composition of three species representative of key freshwater macroalgae genera across a range of cultivation conditions. We then selected a primary target species and assessed its competitive ability against other species over a range of stocking densities. *Oedogonium* had the highest productivity (8.0 g ash free dry weight m^−2^ day^−1^), lowest ash content (3–8%), lowest water content (fresh weigh: dry weight ratio of 3.4), highest carbon content (45%) and highest bioenergy potential (higher heating value 20 MJ/kg) compared to *Cladophora* and *Spirogyra.* The higher productivity of *Oedogonium* relative to *Cladophora* and *Spirogyra* was consistent when algae were cultured with and without the addition of CO_2_ across three aeration treatments. Therefore, *Oedogonium* was selected as our primary target species. The competitive ability of *Oedogonium* was assessed by growing it in bi-cultures and polycultures with *Cladophora* and *Spirogyra* over a range of stocking densities. Cultures were initially stocked with equal proportions of each species, but after three weeks of growth the proportion of *Oedogonium* had increased to at least 96% (±7 S.E.) in *Oedogonium-Spirogyra* bi-cultures, 86% (±16 S.E.) in *Oedogonium-Cladophora* bi-cultures and 82% (±18 S.E.) in polycultures. The high productivity, bioenergy potential and competitive dominance of *Oedogonium* make this species an ideal freshwater macroalgal target for large-scale production and a valuable biomass source for bioenergy applications. These results demonstrate that freshwater macroalgae are thus far an under-utilised feedstock with much potential for biomass applications.

## Introduction

Macroalgae have diverse biomass applications as a source of food and hydrocolloids [1], as fertiliser and soil conditioners [2], and more recently as a targets for a broad range of biofuels [3]–[6]. The majority of these applications utilise marine macroalgae (seaweed) and no significant production of freshwater macroalgae exists. However, this is likely to change. Demand for biofuels is increasing and there is widespread recognition that a viable biofuels industry must be based around feedstocks that use minimal amounts of freshwater and commercial fertilisers and do not directly compete with food production [7]–[9]. Macroalgae satisfy all three requirements when cultivated in industrial waste water and their bioenergy potential is favourable (e.g. [6]). Concurrently, as freshwater ecosystems become threatened by industrial pollution and excessive nutrient loading [10], the use of live algae to remove pollutants and excess nutrients from water – algal bioremediation – is receiving increased attention due to the low costs of implementation compared to alternative physico-chemical treatment methods [11] and the ability to directly grow algae in waste waters [12]–[14]. As most major industries and waste water streams are based around freshwater rather than saltwater (e.g. agriculture, mineral processing, energy production, municipal waste), increasing development of both an algal biofuels industry and algal bioremediation is likely to result in increased cultivation of freshwater macroalgae, supported by concepts derived from a mature seaweed industry.

In contrast to seaweed, target species of freshwater macroalgae for intensive mono-culture are yet to be identified. Several key characteristics are desirable in a target species, irrespective of the biomass application. As most industrial applications and potential end-product uses of macroalgae require large amounts of biomass, it is essential for target species to have high “areal” biomass productivity, expressed as grams of dry weight per unit area (m^2^) per time (day) [15],[16]. Additionally, species should be able to grow across a wide range of conditions with the aim of year round production in open culture systems and controlled water motion to maximise photosynthetic yields [16],[17]. Target species should therefore be competitively dominant to prevent cultures becoming overgrown by nuisance species, a problem that has plagued long-term production of algal monocultures [17]. Finally, low variation in biochemical composition over a range of cultivation conditions is also desirable to ensure a consistent source of biomass for end-product applications. This is particularly the case for biofuel applications, where the productivity of the organic component of the biomass is paramount to bioenergy potential which is typically expressed as the higher heating value in MJ/kg.

Therefore, as a first step to identifying target species of freshwater macroalgae for biomass applications, we compared the productivity, growth and biochemical composition of three species representative of key freshwater macroalgae genera across a range of cultivation conditions representative of intensive culture systems. We then selected a primary target species and assessed its competitive ability against other species over a range of stocking densities. Our overall objective was to identify a freshwater macroalga suitable for large scale cultivation in industrial waste water streams to provide biomass for a range of end-product applications. To do this we focus on filamentous species of freshwater macroalgae from the genera *Cladophora, Spirogyra* and *Oedogonium.* These genera were chosen as they all have broad geographic distributions, are representative of the macroalgae available in many freshwater environments, have rapid growth and can become pest species when nutrient levels are high [18],[19].

## Methods

### Study Species

**Figure 1:**
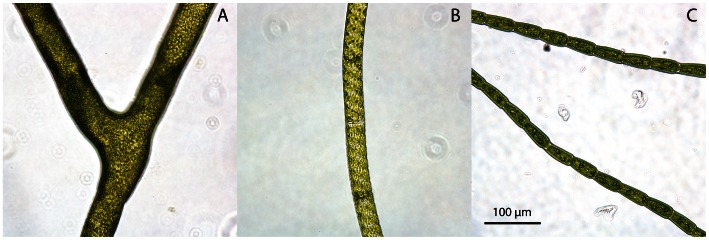
Study species. The three study species - *Cladophora* (A), *Spirogyra* (B) and *Oedogonium* (C).

This study compared three types of freshwater macroalgae from the genera *Cladophora, Spirogyra* and *Oedogonium* (Fig. 1). *Cladophora* species are branching algae with reasonably large filaments (cell diameter 66–133 µm) that commonly form thick mats and turfs. *Spirogyra* species have intermediate sized unbranched filaments (cell diameter 65–88 µm) and typically form dense floating mats. *Oedogonium* species have very fine unbranched filaments (cell diameter 18–32 µm) and commonly grow attached to aquatic vegetation, but can also form floating mats. Both *Cladophora* and *Spirogyra* are late successional species that are commonly found in established macroalgal communities [20]. Species were identified using taxonomic keys [21],[22] and subsequently with DNA sequencing analysis (Supporting information, Text S1). However, identification was only possible to genus level using taxonomic keys as algae lacked species-specific defining characteristics, and DNA sequencing failed to identify unique species (hereafter we refer to genera only: *Cladophora, Spirogyra* and *Oedogonium*). For *Oedogonium*, 3 of the 4 most closely related species from DNA sequencing analysis are located in a clade formed by the monoecious taxa (Clade B [23]), suggesting that our *Oedogonium* species also falls within this clade (Table S1). All new genetic sequences were deposited in GenBank (Accession numbers: KC701472, KC701473, KC701474).

### Culture Methods

Stock cultures of the three species were collected from outdoor ponds at the Baramundi Fishing Farm Townsville and Good Fortune Bay Fisheries Ltd Kelso. Permission was obtained from owners to collect algae from these sites. Stock cultures were grown in a greenhouse in 60 L plastic buckets with ambient natural light at the Marine and Aquaculture Research Facility Unit, James Cook University. Cultures were provided with aeration by a continuous stream of air entering the cultures through multiple inlets around the base of the buckets. Additional dissolved inorganic carbon was provided to some cultures in the form of CO_2_ intermittently pulsed directly into the culture water though an airstone between the hours of 8 am and 4 pm. Culture water was enriched (0.1 g L^−1^) with MAF growth medium (Manutech Pty Ltd, 13.4% N, 1.4% P), which was non-limiting in nitrogen and phosphorus for our culture system (Text S2, Table S2). Stock cultures were maintained for a period of at least four weeks prior to the start of each experiment to allow acclimation to the culture system and ensure that all algae were pre-exposed to identical conditions. All experimental replicates were maintained in 20 L plastic buckets under the same conditions and ambient light. Water temperature and pH were measured daily in each culture. To simulate environments with low water flow that the algae would likely be grown in if cultured in industrial waste water (e.g. settlement ponds, ash dams), the water in each culture was partially exchanged twice a week at a rate equating to a 10% replacement of the total water volume per day. The species selection and competition experiments were run two months apart.

### Species Selection Experiment

To determine which species had the highest growth and productivity under a range of different culture conditions, four replicate cultures of each species were grown with and without CO_2_ under each of three aeration treatments (no aeration, low aeration and high aeration). Supplying CO_2_ has been shown to significantly increase algal productivity [16],[24] as it provides additional dissolved inorganic carbon (DIC), which can become limiting under intensive culture conditions [25],[26]. Cultures had an average pH of 8.2 (±2.0 S.D.) for the CO_2_ treatment and 10.5 (±1.5 S.D.) for the treatment without CO_2_. Bottom aeration of macroalgae cultures is proposed to increase areal productivity by generating vertical movement and water turbulence within the culture, exposing stock to optimal light and increasing the flow of nutrients around the algal surface [27]–[29]. Air flow for the low aeration treatment was set as the minimum amount required to keep algae in constant motion (2 L min^−1^). This flow rate was quadrupled for the high aeration treatment (8 L min^−1^). To provide a proxy for the relative level of water movement these different aeration rates provided, dissolution rates of gypsum balls in each aeration treatment were measured. Dissolution rates in the high aeration treatment were approximately double those of the low aeration treatment (high aeration: 0.40 g hour^−1^ (±0.03 S.E), low aeration: 0.21 g hour^−1^ (±0.05 S.E)), indicating that four times as much airflow is required to double water movement in our system. We used a low and high aeration treatment to generate two levels of water movement as increasing water flow and turbulence can enhance productivity and growth [30],[31]. Average water temperature was 27.7°C (±1.6 S.D.) and cultures received an average of 30.9 mol photons m^−2^ day^−1^ (±3.0 S.D.). Cultures were stocked at a rate of 0.5 g fresh weight (FW) L^−1^ and harvested and weighed after 7 days. Biomass samples were taken from each replicate upon harvesting and dried in an oven at 65°C for at least 24 hours to determine fresh weight : dry weight (FW:DW) ratios for each individual replicate for each week of growth. The ash content of each replicate was quantified by combusting a 500 mg subsample of dried biomass at 550°C in a muffle furnace until constant weight was reached. Following harvesting, stocking density was reset back to 0.5 g FW L^−1^ by removing excess biomass in each culture. The experiment was run for a total of three weeks, providing for three harvests.

Both ash free dry weight (AFDW) productivity (g AFDW m^−2^ day^−1^) and specific growth rate (SGR) were calculated for each replicate for each week as each provide different metrics. AFDW productivity is a measure of the amount of organic biomass produced per unit area, whereas SGR provides information on the relative growth rates of individuals within the culture. AFDW was calculated using the equation P = {[(B_f_ – B_i_)/FW:DW ]*(1-ash) }/A/T, where *B_f_* and *B_I_* are the final and initial algal biomasses (g), *FW:DW* is the fresh weight to dry weight ratio, *ash* is the proportional ash content of the dried biomass, *A* is the area (m^2^) of our culture tanks and *T* is the number of days in culture. Specific growth rate was calculated using the equation SGR (% day^−1^) = Ln(B_f_/B_i_)/T*100, where *B_f_* and *B_I_* are the final and initial algal biomasses (g) and *T* is the number of days in culture. Permutational analyses of variance (PERMANOVAs) were used to analyse the effect of week, species, CO_2_ and aeration on AFDW productivity, specific growth rate, FW:DW ratios and ash content (Table S3).

Biomass samples from replicates of each species cultured with and without CO_2_ at the high aeration level from week 3 were analysed for carbon, hydrogen, oxygen, nitrogen and sulphur (ultimate analysis) (OEA Laboratories UK). To quantify the suitability of biomass as a potential biofuel the higher heating value (HHV) was calculated for each sample. The HHV is based on the elemental composition of the biomass and is a measure of the amount of energy stored within. The HHV was calculated using the equation HHV (MJ/kg)  = 0.3491*C +1.1783*H +0.1005*S –0.1034*O –0.0151*N –0.0211*ash, where *C, H, S, O, N* and *ash* are the carbon, hydrogen, sulphur, oxygen, nitrogen and ash mass percentages of the algae on a dry basis [32].

### Competition Experiment

*Oedogonium* was selected as our target species as it had the highest AFDW culture productivity in five of the six aeration and CO_2_ treatment combinations and the most favourable biochemical composition for end-product applications (see Results and Discussion). To investigate the competitive ability of this species, *Oedogonium–Cladophora* and *Oedogonium-Spirogyra* bi-cultures and a polyculture of all three species were grown at each of three different stocking densities (total densities of 0.25 g FW L^−1^, 0.5 g FW L^−1^, 1 g FW L^−1^). Three replicate cultures of each treatment were established with equal quantities of FW biomass of each species summed to each stocking density. Cultures were grown under high aeration with CO_2_ as *Oedogonium* AFDW productivity was highest under these conditions in the first experiment (see Results and Discussion). Three replicate *Oedogonium* monocultures were also established at each of the three stocking densities as controls. Cultures had an average pH of 9.7 (±0.2 S.D.), average water temperature was 30.1°C (±1.8 S.D.), and cultures received an average of 35.5 mol photons m^−2^ day^−1^ (±3.7 S.D.). Cultures were harvested and weighed after 7 days and a biomass sample was taken from each replicate. Individual FW:DW ratios and ash contents were calculated for each replicate as described above. To estimate the proportional composition of species in all bi-culture and polyculture treatments a biomass sample of 0.4 g FW was sub-sampled from each replicate and suspended in 200 mL dechlorinated water prior to being fixed in Lugols solution (1%). Subsequently, ten replicate sub-samples of each biomass sample were photographed under a dissecting microscope and the proportional species composition calculated by placing a 100-point grid over each photo and summing the number of grid points directly overlying each species. Following harvesting, stocking density was reset back to the original treatment level by removing excess biomass. However, the proportional composition of each species in culture was not reset back to equal levels to quantify the on-going change in species competition (dominance) over time. The experiment was re-run for a further two weeks, providing for a total of three harvests.

Total AFDW productivity was calculated for each replicate for each week as described above. To evaluate competition, specific growth rates were calculated for each replicate for *Oedogonium* only, using the formula above where *B_f_* and *B_I_* are the final and initial biomasses of *Oedogonium* within each culture. *B_f_* was calculated by multiplying the total final FW biomass of each replicate by the proportional composition of *Oedogonium* in that replicate. In week 1 *B_I_* was calculated as half or one third of the total initial biomass stocked into bi-cultures and polycultures respectively; in weeks 2 and 3, *B_I_* was calculated by multiplying the total initial FW biomass by the proportional composition of *Oedogonium* in each replicate in the preceding week. Multivariate PERMANOVAs were used to analyse the effect of competition and density on total AFDW productivity, *Oedogonium* specific growth rates and the proportional composition of *Oedogonium* in bi-cultures and polycultures over the three week experiment (Table S4).

## Results and Discussion

### Species Selection Experiment

**Figure 2:**
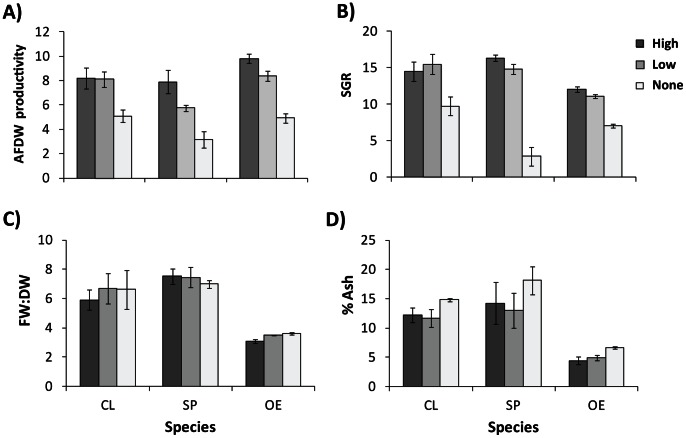
Productivity, growth rates, FW:DW ratios and ash contents of macroalgae cultures. Mean (±S.E.) ash-free dry weight productivity (g m^−2^ day^−1^) (A), specific growth rate (SGR, % day) (B), FW:DW ratio (C) and ash content (D) of three macroalgae grown under three aeration levels. CL: *Cladophora*; SP: *Spirogyra*; OE: *Oedogonium*. Data are pooled across CO_2_ treatments. Standard errors are calculated as the variation in means between the three weeks of the experiment (n  = 3).

Productivity, as determined by AFDW, varied significantly between the three species (Fig. 2a). *Oedogonium* was the most productive species across all treatments when grown under high aeration with CO_2_ (8.0 g AFDW m^−2^ day^−1^) and the productivity of *Oedogonium* was at least 20% greater than that of *Cladophora* and *Spirogyra* in all treatment combinations except when grown with low aeration and no CO_2_ (Table S3). In contrast to productivity as measured by AFDW, specific growth rate was highest across all treatments for *Cladophora* when grown under low aeration with CO_2_ (17.4% day^−1^). In all treatment combinations, *Cladophora* growth rates were at least 30% higher than *Oedogonium* and, with the exception of the no aeration treatment, *Spirogyra* growth rates were at least 20% higher (Fig. 2b; Table S3). Striking differences in the relative position of the three species in AFDW productivity compared to specific growth rate were driven by differences in their FW:DW ratios and ash contents. FW:DW ratios varied significantly between species (Fig. 2c; Table S3), with the ratio for *Spirogyra* (7.3±0.22 S.E.) being more than double that of *Oedogonium* (3.4±0.04 S.E.). There were also significant differences in ash content between species (Fig. 2d; Table S3). *Oedogonium* ash contents (3–8%) were less than half those of *Cladophora* (11–16%) and *Spirogyra* (12–19%) in every individual treatment combination. Consequently, despite slower growth rates, *Oedogonium* cultures produced larger amounts of dried ash-free biomass - the critical measure for the majority of end-product applications, particularly bioenergy. Rapid growth rates are often used as one of the key desirable characteristics when assessing the suitability of algae for large scale cultivation [33]. However, as has been shown for other macroalgae species [34], our results demonstrate that fast growth rates are not necessarily equivalent to high productivity, providing support to previous assertions that culture productivities should not be extrapolated from growth rates obtained in controlled experiments [17].

The key biological attributes of *Oedogonium* that contributed to its higher AFDW productivity - lower ash content and lower FW:DW - are also important considerations in the evaluation of feedstocks for biomass applications. For example, a higher water content (high FW:DW values) means higher inputs are required to obtain dried feedstock, which is necessary if the feedstock is to be transported from point of production to a centralised processing location [35]. Similarly, higher ash contents appear to be correlated with high water contents and may negatively influence bioenergy processes such as hydrothermal liquefaction (HTL) and biogas production due to the concentration of mineral salts at higher levels than traditional lignocellulosic feedstocks [3]. Species differences for bioenergy potential were also reflected in the CHONS analysis and higher heating values (Table 1). *Oedogonium* had the highest carbon content (45%) and correspondingly the best higher heating values (∼20 MJ kg^−1^). These values are comparable to those recorded for terrestrial energy crops of woody plants (16–23 MJ kg^−1^) [36]–[38], confirming that *Oedogonium* biomass has high energy potential and would provide a suitable feedstock for bioenergy applications. Furthermore, the consistently high productivity recorded for *Oedogonium* across a range of conditions (e.g. high/low aeration, with/without CO_2_) implies that this species can be reliably grown in a variety of cultivation systems, and is also compatible with industrial waste water streams to provide algal bioremediation (e.g. [13],[14]).

**Table 1:** Ultimate analysis of macroalgae biomass.

Species	CO_2_ treatment	Ash	C	H	O	N	S	HHV
*Oedogonium*	CO_2_	2.9 (0.2)	45.3 (0.1)	6.7 (0.1)	38.3 (0.9)	3.5 (0.0)	0.0 (0.0)	19.7 (0.2)
	No CO_2_	3.7 (0.5)	45.5 (0.2)	6.9 (0.0)	37.4 (0.6)	3.6 (0.1)	0.1 (0.1)	20.1 (0.1)
								
*Cladophora*	CO_2_	9.5 (0.7)	43.1 (0.3)	6.2 (0.1)	34.5 (0.9)	4.6 (0.2)	0.3 (0.2)	18.6 (0.2)
	No CO_2_	12.1 (2.0)	43.0 (0.5)	6.3 (0.1)	34.3 (1.0)	4.7 (0.1)	0.2 (0.1)	18.6 (0.2)
								
*Spirogyra*	CO_2_	13.5 (2.1)	42.7 (0.5)	6.3 (0.0)	35.4 (1.2)	4.4 (0.1)	0.0 (0.1)	18.3 (0.4)
	No CO_2_	8.7 (0.8)	43.6 (0.1)	6.4 (0.1)	36.8 (0.5)	4.3 (0.1)	0.1 (0.0)	18.7 (0.1)

Ash, ultimate analysis (weight %, on a dry basis) and higher heating value (MJ/kg, on a dry basis) of biomass from three freshwater macroalgae cultured with and without CO_2_. Values are means (±S.E.), n = 4, biomass was sampled at the end of the species selection experiment. Note that *Cladophora* and *Spirogyra* samples were not pure cultures (see Results and Discussion).

Cultivation conditions are clearly important for biomass production as all treatments had variable effects on culture productivity, growth rates, FW:DW ratios and ash content over the three experimental weeks (Table S3). In general, cultures without aeration had lower growth rates and AFDW productivity, and higher ash contents relative to treatments with aeration (Figs. 2a,b,d; Table S3). Variation in both FW:DW ratios and ash content was much greater between species than between treatments within each species, and both *Cladophora* and *Spirogyra* cultures with high FW:DW ratios consistently had high ash contents (Figs. 2c,d; Table S3). Notably these same species had the highest growth rates and lowest AFDW productivities. In contrast to recent research showing that CO_2_ can have pronounced effects on *Oedogonium* productivity [39], CO_2_ had no effect on AFDW productivity or growth rate in the current study (Table S3), suggesting that cultures without additional CO_2_ were not limited by the availability of dissolved inorganic carbon. However, as CO_2_ was directly bubbled into cultures as a gas and not dissolved in the water, it is also possible that a large proportion of the CO_2_ added to cultures was lost to the atmosphere through off gassing [24], resulting in minimal differences in the amount of dissolved inorganic carbon supplied to cultures. Some of the variability in the experiment for *Cladophora* and *Spirogyra* was driven by contamination of cultures with other species (predominantly *Hydrodictyon* species and *Stigeoclonium* species), resulting from the growth of dormant spores or small contaminant filaments in the biomass when it was first collected. Analysis of the biomass composition at the end of the experiment indicated that contamination was ∼80% in *Cladophora* cultures and ∼30% in *Spirogyra* cultures, inferring that it will be difficult to maintain monocultures of these species over extended periods.

### Competition Experiment

**Figure 3:**
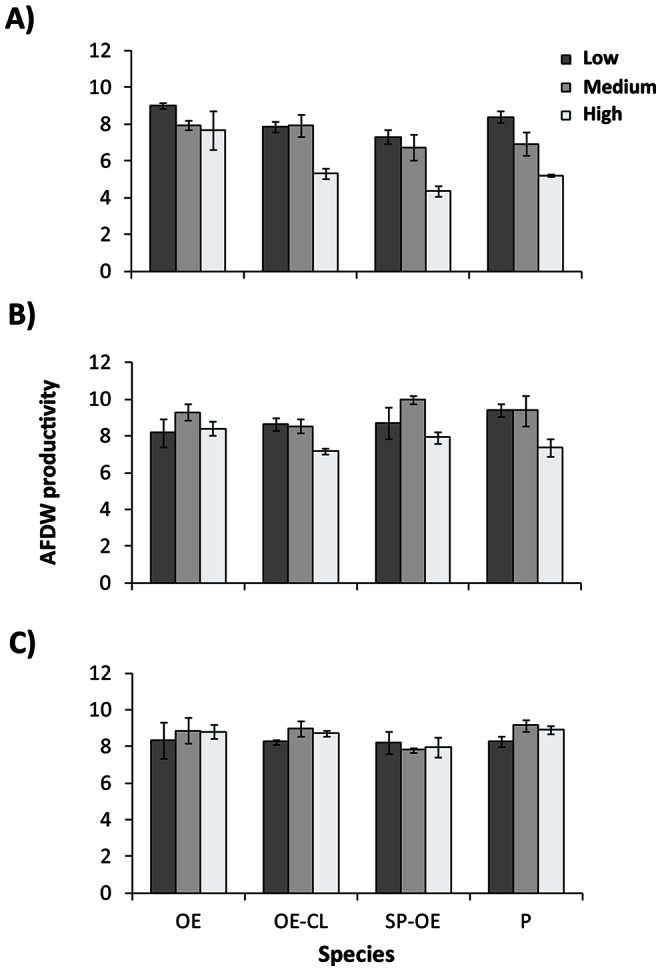
AFDW productivity of mixed species cultures in competition experiment. Mean (±S.E.) total ash free dry weight productivity (g m^−2^ day^−1^) of monoculture, bi-culture and polyculture combinations of three macroalgae grown under three stocking densities (low, medium, and high) in A) Week 1, B) Week 2 and C) Week 3 of the competition experiment. OE: *Oedogonium* monoculture (control); CL-OE: *Cladophora – Oedogonium* bi-culture; SP-OE: *Spirogyra – Oedogonium* bi-culture; P: Polyculture of all three species.

**Figure 4:**
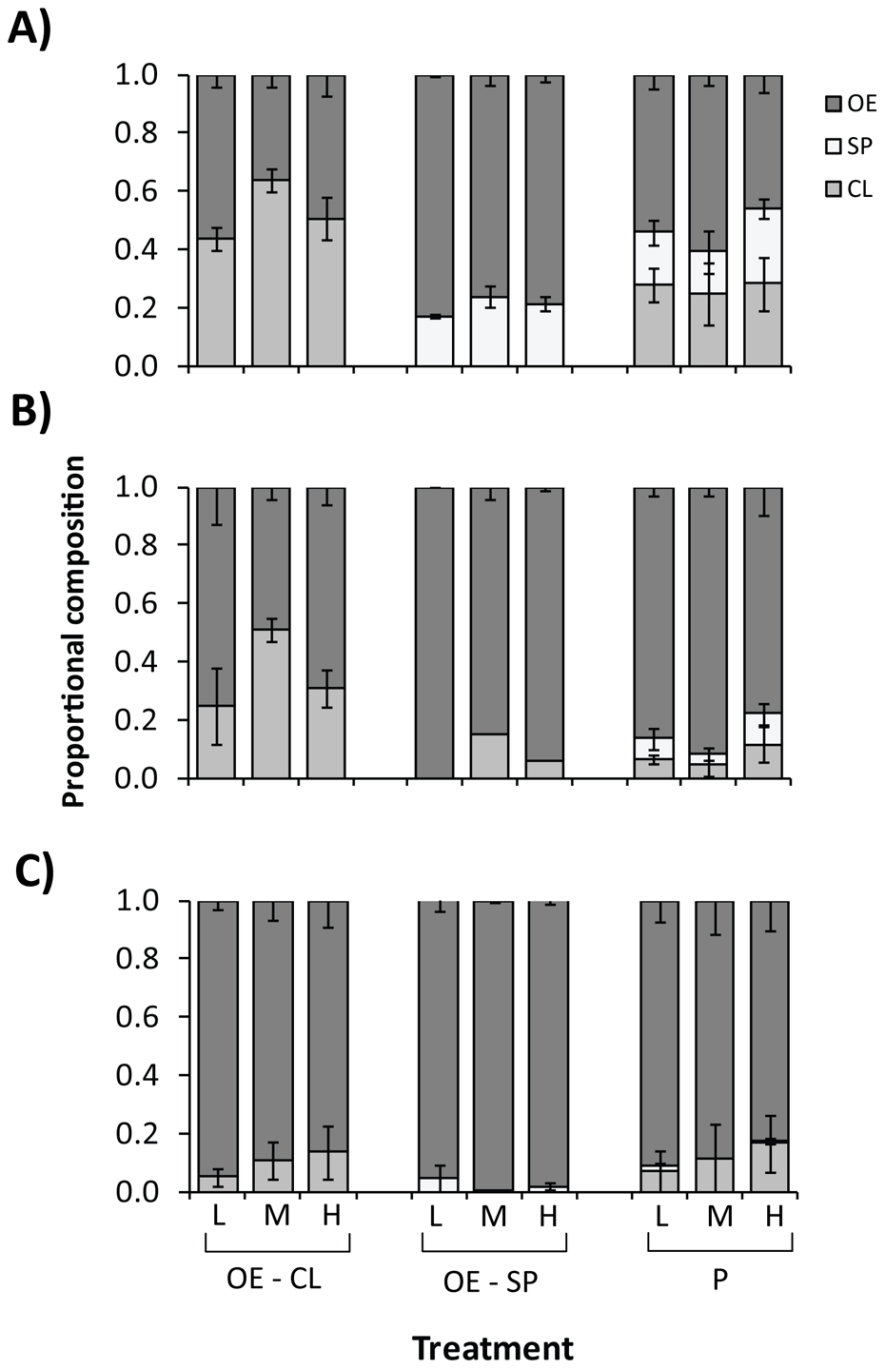
Proportional composition of mixed species cultures. Mean (±S.E.) proportional species composition of bi-culture and polyculture combinations of three macroalgae grown under three stocking densities (low, medium, and high) in A) Week 1, B) Week 2 and C) Week 3 of the competition experiment. Species abbreviations follow Fig. 3.

In general, the AFDW productivity of mixed species cultures was at least 10% lower than *Oedogonium* monocultures in the first week of the competition experiment, but there were no differences between cultures in the third week (Fig. 3; Table S4). Changes in culture AFDW productivities between weeks reflect increases in the relative proportions of *Oedogonium* in bi-cultures and polycultures over the course of the three-week experiment (Fig. 4). Although bi-cultures and polycultures were initially stocked with equal proportions of each species, by the end of the third week the proportion of *Oedogonium* in mixed species cultures was not significantly different (Table S4) and had increased to at least 96% (±7 S.E.) in *Oedogonium-Spirogyra* bi-cultures, 86% (±16 S.E.) in *Oedogonium-Cladophora* bi-cultures and 82% (±18 S.E.) in polycultures. These results clearly demonstrate that *Oedogonium* is competitively dominant and unlikely to become contaminated by other non-target macroalgae species when cultured in “open” systems, providing opportunity for high flow and water exchanges to maximise productivities [30],[31].

When selecting algal species for cultivation, fast growth rates are expected to provide a competitive advantage [33]. Yet in contrast to this expectation, the competitively dominant *Oedogonium* had the lowest growth rate of monocultures of all three species in the species selection experiment. However, in the first week of the competition experiment, growth rates of *Oedogonium* were up to 50% higher in mixed species cultures compared to the monoculture. For example, specific growth rates of *Oedogonium* were 12.2% per day (±0.2 S.E.) in the *Spirogyra-Oedogonium* bi-culture under high stocking density, but only 8.1% (±0.8 S.E.) per day in the *Oedogonium* monoculture. These results suggest that *Oedogonium* may increase growth rate as a competitive response to the presence of other species. Regardless, these results demonstrate that inferring competitive abilities based on the growth rates of species in monoculture can be misleading, and likewise inferring bioenergy potential from growth rates could lead to erroneous conclusions about feedstock quality.

The strong competitive response of *Oedogonium* was unaffected by the total stocking density of cultures, with all cultures arriving at greater than 80% *Oedogonium* at the end of the experiment regardless of stocking density treatment (Fig. 3). Similarly, by the third week of the experiment when all mixed species cultures were dominated by *Oedogonium, s*tocking density had negligible effects on AFDW productivity (Fig. 2; Table S4). In contrast, *Oedogonium* growth rates were significantly higher in the low stocking density treatment (23.4% day^−1^±0.8 S.E.) compared to the medium (16.2% day^−1^±1.0 S.E.) and high (9.8% day^−1^±0.8 S.E.) stocking density treatments across all species combinations (Table S4). Macroalgae productivity is generally higher at higher stocking densities [40],[41]; although this is not always the case (e.g. [42]) and optimal densities can vary between species [43]. Our results suggest that initially stocking *Oedogonium* cultures at low densities (0.25 g L^−1^) and harvesting over longer time periods would result in similar productivity to that achieved by stocking cultures at high densities (1 g L^−1^) and harvesting frequently. This could minimise operational costs associated with harvesting, an important consideration of any aquaculture operation.

### Conclusions

For the first time, this study compares the productivity, growth and biochemical composition of freshwater macroalgae in order to identify target species for intensive single species cultivation. *Oedogonium* had the highest AFDW productivity and a consistent biochemical composition, with a high carbon content and bioenergy potential across a range of cultivation conditions. Moreover, *Oedogonium* was competitively dominant in mixed species cultures and quickly overgrew other species within weeks. *Oedogonium* is a cosmopolitan algal genus with a broad geographical distribution. In combination, these factors make *Oedogonium* an ideal freshwater macroalgal target for large-scale production and as a biomass source for bioenergy applications. Our results show that green freshwater macroalgae have much potential for biomass applications but are thus far an under-utilised feedstock. They represent a diverse group of algae for which the greatest opportunity appears to be with small filamentous morphologies, such as *Oedogonium*, that are more cryptic than larger, end succession macroalgae that are apparent in algal blooms (e.g. *Cladophora, Spirogyra*).

## Supporting Information

Table S1 **GenBank accession numbers and results of BLAST searches for** ***Oedogonium*** **sequences at four DNA barcode markers.**
(DOCX)

Table S2 **Water nutrient concentrations and productivity of three macroalgae species in nutrient limitation pilot experiments.**
(DOCX)

Table S3 **Results of full factorial permutational analyses of variance (PERMANOVAs) testing the effects of week, species, CO_2_ and aeration on productivity as AFDW, specific growth rate, FW:DW ratios and ash content of cultures in the species selection experiment.**
(DOCX)

Table S4 **Results of full factorial multivariate permutational analyses of variance (PERMANOVAs) testing the effects of competition and density on productivity as AFDW, proportional composition of** ***Oedogonium*** **and specific growth rate of** ***Oedogonium*** **in cultures in the competition experiment.**
(DOCX)

Text S1 **DNA sequencing identification of algae.**
(DOCX)

Text S2 **Pilot experiments to test for nutrient limitation.**
(DOCX)
