# Supplementary material for: Selecting Reliable and Robust Freshwater Macroalgae for Biomass Applications
Source: PLoS One. 2013 May 22;8(5):e64168. doi: 10.1371/journal.pone.0064168 (PMC3661442; doi:10.1371/journal.pone.0064168)
Supplement: Table S1 — GenBank accession numbers and results of BLAST searches for Oedogonium sequences at four DNA barcode markers. (DOCX) [file pone.0064168.s001.docx]

**Table S1**

GenBank accession numbers and results of BLAST searches for *Oedogonium* sequences at four DNA barcode markers. Species with the highest matched sequence identity for each marker are reported. Identities show the number of identical base pairs between sequences out of the total sequence length; the bit score measures the similarity between the queried sequence and the matched sequence, with higher values indicating greater similarities; the E value represents the number of alignments you would expect to find by chance that have the same score as the alignment you are looking at.

| **Marker** | **Accession number** | **Species** | **Identities** | **Bit score** | **E value** |
| --- | --- | --- | --- | --- | --- |
| LSU | KC701472 | *Oedogonium nodulosum* | 605/615 (98%) | 1081 | 0.0 |
| ITS | KC701473 | *Oedogonium globosum* | 575/674 (85%) | 664 | 0.0 |
| rbcL3’ | KC701474 | *Oedogonium sp.* | 654/665 (99%) | 1168 | 0.0 |
| rbcL5’ | KC701474 | *Oedogonium cardiacum* | 520/527 (99%) | 935 | 0.0 |
