# Supplementary material for: Selecting Reliable and Robust Freshwater Macroalgae for Biomass Applications
Source: PLoS One. 2013 May 22;8(5):e64168. doi: 10.1371/journal.pone.0064168 (PMC3661442; doi:10.1371/journal.pone.0064168)
Supplement: Table S2 — Water nutrient concentrations and productivity of three macroalgae species in nutrient limitation pilot experiments. (DOCX) [file pone.0064168.s002.docx]

**Table S2**

Water nutrient concentrations of NO_X_ and Filterable Reactive Phosphorus, and AFDW productivity of three macroalgae grown for two consecutive weeks.

|  | **NO_X_ (µg N/L)** | | **Phosphorus (µg P/L)** | | **Productivity (g AFDW m^-2^ day^-1^)** | |
| --- | --- | --- | --- | --- | --- | --- |
|  | ***Replicate A*** | ***Replicate B*** | ***Replicate A*** | ***Replicate B*** | ***Replicate A*** | ***Replicate B*** |
| *Cladophora* |  |  |  |  |  |  |
| Week 1 | 5058 | 7954 | 21 | 543 | 9.6 | 6.2 |
| Week 2 | 6372 | 1690 | 7 | 4 | 4.6 | 5.8 |
| *Oedogonium* |  |  |  |  |  |  |
| Week 1 | 5070 | 5151 | 8 | 6 | 9.2 | 9.2 |
| Week 2 | 2318 | 4282 | 3 | 3 | 8.4 | 6.4 |
| *Spirogyra* |  |  |  |  |  |  |
| Week 1 | 7956 | 6792 | 177 | 126 | 5.3 | 4 |
| Week 2 | 4986 | 1987 | 4 | 4 | 6.9 | 7.8 |
