# Supplementary material for: Selecting Reliable and Robust Freshwater Macroalgae for Biomass Applications
Source: PLoS One. 2013 May 22;8(5):e64168. doi: 10.1371/journal.pone.0064168 (PMC3661442; doi:10.1371/journal.pone.0064168)
Supplement: Table S3 — Results of full factorial permutational analyses of variance (PERMANOVAs) testing the effects of week, species, CO2 and aeration on productivity as AFDW, specific growth rate, FW:DW ratios and ash content of cultures in the species selection experiment. (DOCX) [file pone.0064168.s003.docx]

**Table S3**

Results of full factorial permutational analyses of variance (PERMANOVAs) testing the effects of week (We, random factor), species (Sp), CO_2_ (CO) and aeration (Ae) (all fixed factors) on AFDW productivity (AFDW), specific growth rate (SGR), FW:DW ratios (FW:DW) and ash content (Ash) of cultures in the species selection experiment. Analyses were conducted in Primer v6 (Primer-E Ltd, UK) using Bray-Curtis dissimilarities on fourth root transformed data and 999 unrestricted permutations of raw data [47]. Pseudo F (F) and P values are presented, significant terms shown in bold.

|  |  | **AFDW** | | **SGR** | | **FW:DW** | | **Ash** | |
| --- | --- | --- | --- | --- | --- | --- | --- | --- | --- |
| **Source** | **df** | **F** | **P** | **F** | **P** | **F** | **P** | **F** | **P** |
| Sp | 2 | **48.9** | **0.005** | **20.8** | **0.001** | **12.4** | **0.027** | **20.8** | **0.003** |
| CO | 1 | 1.2 | 0.365 | 0.9 | 0.433 | 3.0 | 0.204 | 0.9 | 0.459 |
| Ae | 2 | **400.0** | **0.003** | **94.5** | **0.001** | 1.1 | 0.430 | **94.5** | **0.001** |
| We | 2 | 1.4 | 0.241 | 1.2 | 0.358 | 0.4 | 0.949 | 1.2 | 0.348 |
| Sp x CO | 2 | 0.7 | 0.563 | 0.5 | 0.684 | 0.3 | 0.750 | 0.5 | 0.661 |
| Sp x Ae | 4 | **48.1** | **<0.001** | **35.0** | **0.001** | **4.0** | **0.047** | **35.0** | **0.001** |
| Sp x We | 4 | 1.3 | 0.332 | 1.1 | 0.374 | **4.8** | **0.009** | 1.1 | 0.380 |
| CO x Ae | 2 | 0.5 | 0.623 | 1.2 | 0.392 | 4.1 | 0.097 | 1.2 | 0.383 |
| CO x We | 2 | 0.7 | 0.720 | 1.0 | 0.519 | 0.3 | 0.931 | 1.0 | 0.537 |
| Ae x We | 4 | 0.7 | 0.744 | 1.1 | 0.403 | 1.0 | 0.518 | 1.1 | 0.400 |
| Sp x CO x Ae | 4 | 0.3 | 0.872 | 0.4 | 0.850 | 3.6 | 0.051 | 0.4 | 0.850 |
| Sp x CO x We | 4 | 0.9 | 0.500 | 0.9 | 0.523 | 2.4 | 0.150 | 0.9 | 0.504 |
| Sp x Ae x We | 8 | 0.2 | 0.990 | 0.2 | 0.994 | 0.2 | 0.967 | 0.2 | 0.998 |
| CO x Ae x We | 4 | 1.4 | 0.313 | 1.0 | 0.500 | 1.7 | 0.201 | 1.0 | 0.507 |
| Sp x CO x Ae x We | 8 | 1.8 | 0.057 | **2.1** | **0.028** | **2.4** | **0.020** | **2.1** | **0.034** |

***See main article for references***
