# Supplementary material for: Selecting Reliable and Robust Freshwater Macroalgae for Biomass Applications
Source: PLoS One. 2013 May 22;8(5):e64168. doi: 10.1371/journal.pone.0064168 (PMC3661442; doi:10.1371/journal.pone.0064168)
Supplement: Table S4 — Results of full factorial multivariate permutational analyses of variance (PERMANOVAs) testing the effects of competition and density on productivity as AFDW, proportional composition of Oedogonium and specific growth rate of Oedogonium in cultures in the competition experiment. (DOCX) [file pone.0064168.s004.docx]

**Table S4**

Results of full factorial multivariate permutational analyses of variance (PERMANOVAs) testing the effects of competition (Co) and density (De) (both fixed factors) on AFDW productivity (AFDW), proportional composition of Oedogonium (% OE) and specific growth rate of Oedogonium (SGR) in cultures in the competition experiment. Data for each week were treated as separate variables. Analyses were conducted in Primer v6 (Primer-E Ltd, UK) using Bray-Curtis dissimilarities on fourth root transformed data and 999 unrestricted permutations of raw data [47]. Pseudo F (F) and P values are presented, significant terms shown in bold.

|  |  | **AFDW** | | **% OE** | | **SGR** | |
| --- | --- | --- | --- | --- | --- | --- | --- |
| **Source** | **df** | **F** | **P** | **F** | **P** | **F** | **P** |
| Co | 2 | **18.9** | **<0.001** | **23.2** | **<0.001** | **9.6** | **<0.001** |
| De | 2 | 2.5 | 0.072 | **3.2** | **0.029** | 1.2 | 0.312 |
| Co x De | 4 | 1.0 | 0.456 | 1.4 | 0.241 | 0.7 | 0.653 |

***See main article for references***
