# Supplementary material for: Selecting Reliable and Robust Freshwater Macroalgae for Biomass Applications
Source: PLoS One. 2013 May 22;8(5):e64168. doi: 10.1371/journal.pone.0064168 (PMC3661442; doi:10.1371/journal.pone.0064168)
Supplement: Text S1 — DNA sequencing identification of algae. (DOCX) [file pone.0064168.s005.docx]

**Text S1**

**DNA sequencing identification of algae**

Total DNA was extracted from two dried samples each of *Cladophora, Spirogyra* and *Oeodognium* using a Qiagen DNEasy Plant Mini Kit following the manufacturer’s instructions. DNA was amplified at the DNA barcoding markers rbcL3’, rbcL5’, LSU and ITS using the primers detailed in Saunders & Kucera [44]. These makers were chosen based on their ability to successfully discriminate between species of chlorophytan green algae [44,45]. Polymerase chain reaction (PCR) amplifications were performed in a 25 µL reaction mixture containing 1.5 U of MyTaq HS DNA polymerase (Bioline^TM^), 5 x MyTaq reaction buffer, 0.4 µM each primer, and 1 µL of genomic DNA (25 – 30 ng). Amplification was performed on a BioRad C1000 Thermal Cycler (cycling parameters: 2 min at 94 °C, 30 cycles of 30 s denaturing at 94 °C, 45 s annealing at 48 - 56 °C, 60 s extension at 72 °C, and a final extension at 72 °C for 10 min). PCR products were column purified using Sephadex G-25 resin and sequenced in both directions by the Australian Genome Research Facility, Brisbane, Australia. Sequences were edited using Sequencher v4.5 (Gene Codes Corporation, Ann Arbor, MI, USA), then sequence similarity searches were performed using a nucleotide BLAST search in GenBank ([http://www.ncbi.nlm.nih.gov/BLAST/](http://www.sciencedirect.com/science?_ob=RedirectURL&_method=externObjLink&_locator=url&_issn=03790738&_origin=article&_zone=art_page&_plusSign=%2B&_targetURL=http%253A%252F%252Fwww.ncbi.nlm.nih.gov%252FBLAST%252F)) and a Barcode of Life Datasystems (BOLD) search (<http://www.barcodinglife.org/>). All sequences were submitted to NCBI GenBank (Supporting information, Table S1).

*Cladophora* DNA failed to amplify at all four markers and *Spirogyra* DNA failed to amplify at the LSU marker, despite performing PCRs across a range of annealing temperatures (48 – 56°C) and DNA concentrations (10 – 40 ng). All other amplification reactions were successful, however only *Oedogonium* sequences were analysed as all *Spirogyra* sequences were unreadable. Both a GenBank BLAST search and a BOLD search failed to provide a clear species identification for our *Oedogonium* strain. There were no identical matches for sequences generated by any of the four barcode markers to sequences in either database, and the species showing the highest matched sequence identity was different for each marker (Table 1). However 3 of the 4 most closely related species are located in a clade formed by the monoecious *Oedogonium* taxa (Clade B [23]), suggesting that our strain also falls within this clade.

***See main article for references***
