# Supplementary material for: Selecting Reliable and Robust Freshwater Macroalgae for Biomass Applications
Source: PLoS One. 2013 May 22;8(5):e64168. doi: 10.1371/journal.pone.0064168 (PMC3661442; doi:10.1371/journal.pone.0064168)
Supplement: Text S2 — Pilot experiments to test for nutrient limitation. (DOCX) [file pone.0064168.s006.docx]

**Text S2**

**Pilot experiments to test for nutrient limitation**

To determine whether algal productivity was limited by the availability of nitrogen and phosphorus in the MAF growth medium, two replicate cultures of each species (*Cladophora, Spirogyra* and *Oedogonium*) were grown in filtered freshwater enriched with MAF medium at a rate of 0.1 g L^-1^ and with aeration (8 L min^-1^) and CO_2_. Cultures were stocked at rate of 0.5g fresh weight L^-1^ and the water in each culture was partially exchanged twice a week at a rate equating to a 10% turnover of the total water volume per day. Cultures were harvested once per week and AFDW productivity calculated for each replicate (as described in methods). Upon harvesting, water samples were taken from each replicate and analysed externally (Australian Centre for Tropical and Freshwater Research, James Cook University) for filterable reactive phosphorus and NO_x_ . The experiment was run for a period of two weeks.

Nutrient concentrations in culture water declined during the experiment (Supporting information, Table S2), however concentrations of NO_x_ were still at reasonable levels (1690 – 6372 µg L^-1^) at the end of the second week. In contrast, concentrations of filterable reactive phosphorus were extremely low (<10 µg L^-^1) in three of the six replicates after the first week, and in all six replicates after the second week. AFDW yields of all *Cladophora* and *Oedogonium* replicates were lower in week 2 compared to week 1, suggesting that productivity may have been limited by phosphorus availability.

To determine whether supplying additional phosphorus resulted in higher productivity, three replicate cultures of each species were grown at each of three different phosphorus treatments (P, 2P, 4P), with aeration (8 L min^-1^) and CO_2_. Experimental conditions were as described above, but in addition to MAF medium, phosphorus in the form of NaH_2_PO_4_ was added to the 2P and 4P treatments to give a total concentration equivalent to two times (2P) and four times (4P) the concentration of phosphate in the MAF medium. Cultures were stocked at rate of 0.5g fresh weight L^-1^ and allowed to grow for 7 days. After this time total biomass in each replicate culture was harvested and weighed and AFDW productivity was calculated.

There were no significant differences in AFDW yield between the three phosphorus treatments for any of the three species (One way ANOVA, Cladophora: F_2,6_=0.83, p=0.48; Oedogonium: F_2,6_=0.04, p=0.96; Spriogyra: F_2,6_=1.41, p=0.32). These results suggest that productivity is not limited by phosphorus availability and the extremely low phosphorus concentrations in water samples were the results of luxury uptake [46].

***See main article for references***
